# Innovative AC Electrospinning and Characterization of Nanofibers Comprised of Polyvinyl Alcohol and Dacarbazine for Solid State Drug Delivery of Cancer Therapeutic

**DOI:** 10.1007/s12668-026-02630-5

**Published:** 2026-05-30

**Authors:** Rachel E. Murphy, Yongzhe Yan, Jadyn Parker, Subhayu Sen, W. Anthony Brayer, Haibin Ning

**Affiliations:** 1 Department of Biomedical Engineering - Neuroengineering, The University of Alabama at Birmingham, Birmingham, AL 35294 USA; 2 TruSpin Nanomaterial Innovation, Birmingham, AL 35203 USA; 3 Department of Mechanical and Materials Engineering, Materials Processing and Applications Development Center, The University of Alabama at Birmingham, Birmingham, AL 35294 USA; 4 Universities Space Research Association, NASA Marshall Space Flight Center, Huntsville, AL 35758 USA

**Keywords:** Alternating current electrospinning, Nanofibers, Dacarbazine, Polyvinyl alcohol, Chemotherapeutic

## Abstract

Dacarbazine (DTIC) is an alkylating chemotherapeutic agent with demonstrated anti-tumor activity but limited utility for intracranial applications due to its chemical instability, short systemic half-life, and inability to cross the blood-brain barrier. Strategies that stabilize DTIC in the solid form for localized delivery therefore are of significant interest due to the material challenges. In this study, nanofibers with various polyvinyl alcohol (PVA)-to-dacarbazine ratios ranging from 2.5:1, 5:1, 7.5:1, to 10:1 were developed using alternating-current electrospinning (ACES) at an extremely high production rate. Their structural and physiochemical properties were systematically characterized to assess the feasibility of this approach using different characterization methods such as X-ray diffraction (XRD), Fourier-transform infrared (FT-IR) spectroscopy, differential scanning calorimetry (DSC), scanning electron microscopy (SEM) and transmission electron microscopy (TEM). Additionally, the encapsulation efficiency (EE) and the drug release profile was evaluated using Ultraviolet-visible spectroscopy (UV-Vis). The characterization results show that nanofibers obtained using the ACES approach have good DTIC dispersion in the PVA matrix and the DTIC remains individualistic in the nanofiber. These results collectively demonstrate that ACES enables the incorporation of dacarbazine into PVA nanofibers while preserving favorable fiber morphology and promoting solid-state stabilization. This work shows the novelty in the application of ACES to fabricate dacarbazine-loaded PVA nanofibers with controlled morphology and solid-state drug stabilization. It also establishes a foundational materials platform for localized dacarbazine delivery and providing a basis for future studies focused on drug release behavior, stability, and therapeutic application.

## Introduction

Dacarbazine (DTIC) is an alkylating chemotherapeutic agent widely used in the treatment of malignant melanoma, Hodgkin’s lymphoma, and other solid tumors [1–5]. As a prodrug, DTIC undergoes hepatic cytochrome P450-mediated activation to form its cytotoxic methylating species [4, 6]. Even though other anti-cancer therapeutics have come on the market since dacarbazine’s market introduction, DTIC remains a component of several anti-tumor treatment plans due to its broad activity and well-characterized pharmacology [4, 7, 8]. More recently, DTIC has been explored for glioblastoma multiforme (GBM), particularly when standard treatments offer limited benefit [9–11]. However, DTIC has a limitation that restricts its intracranial efficacy; DTIC cannot cross the blood-brain barrier (BBB) [12–16]. This prevents therapeutic concentrations from reaching tumor tissue after systemic drug delivery. Additionally, DTIC’s short plasma half-life, rapid degradation, and dose limiting toxicities, including myelosuppression, gastrointestinal side effects, and hepatotoxicity, also limit its clinical use [1, 4, 17–23]. Together, these barriers highlight the need for alternative drug delivery strategies that are capable of achieving stable, localized, therapeutically relevant drug levels at brain tumor sites that also minimize systemic exposure. Localized drug delivery systems that can bypass the BBB entirely are a promising avenue for mitigating these limitations.

Aside from its clinical constraints, DTIC also exhibits several physiochemical and pharmacokinetic challenges. DTIC is highly susceptible to hydrolysis and photolysis which leads to a significant loss of potency during both storage and administration [18, 19, 21, 22]. The drug also displays pH-dependent instability, degrading more readily under physiological conditions than in acidic environments [5, 24–27]. To mitigate these challenges, incorporation of dacarbazine into polymer-based systems has been explored as a strategy to reduce its exposure to aqueous environments and improve stability. In particular, highly hydrolyzed polymers such as polyvinyl alcohol can provide a hydrogen-bonding network and reduced water mobility, which may slow hydrolytic degradation during processing and storage [28–31]. Pharmacokinetically, DTIC’s short plasma half-life, typically 20–50 min, is driven by rapid hepatic clearance [23], which necessitates high systemic doses that exacerbate toxicity [5]. Its hydrophilicity and dependence on hepatic activation further limit its penetration into the brain which in turn renders systemic administration ineffective for GBM [9–11, 32]. Collectively, these properties motivate the development of drug-delivery systems that can stabilize DTIC and enable sustained, localized release.

Polyvinyl alcohol (PVA) is a synthetic, water-soluble polymer with excellent biocompatibility, chemical stability, and film forming capabilities making it widely used in pharmaceutical and biomedical applications [33–35]. Its abundant hydroxyl groups promote hydrogen bonding, contributing to mechanical integrity and facilitating interactions with hydrophilic small-molecule drugs [36]. PVA also exhibits tunable crystallinity, dependent on molecular weight, degree of hydrolysis, and processing conditions, which supports engineered release behavior and solid-state drug stabilization [33, 34, 36–38]. PVA’s favorable rheological and electrostatic properties promote stable plume formation and uniform fiber morphology during electrospinning [36, 39]. In drug delivery applications, PVA nanofibers have successfully encapsulated a wide range of compounds, protected labile drugs, and enabled controlled or sustained release depending on formulation parameters [33, 36, 37, 40, 41]. These features make PVA an appealing polymeric matrix for localized chemotherapeutic delivery.

Beyond these known advantages for using PVA as a drug delivery system, PVA possesses specific characteristics that make it particularly suitable for stabilizing DTIC. Its high density of hydroxyl groups enables extensive hydrogen bonding with embedded molecules which promotes homogeneous molecular dispersion within the polymer matrix [33, 34]. This interaction reduces phase separation, facilitating homogenous solid-dispersion and improving physical stability [3, 36]. Further, hydrolyzed PVA DTIC being encapsulated by the PVA nanofibers also protects the DTIC from hydrolysis and photolysis by reducing water access and limiting exposure to light and oxygen [18, 19, 21]. These effects have been demonstrated in PVA-based systems used to stabilize other chemically labile or photodegradable drugs [3, 36]. Together, these properties support the use of PVA nanofibers as a solid-state platform for incorporating DTIC into stable, localized delivery systems.

Electrospinning is a versatile technique for producing nanofibrous materials with high surface-to-volume ratios, interconnected porosity, and tunable physiochemical behavior [35, 42]. These features make electrospun fibers well suited for drug delivery applications, where fiber structure can be engineered to influence dissolution, diffusion, and degradation pathways [36, 43, 44]. The rapid elongation and solvent evaporation inherent to electrospinning enable incorporation of a wide range of drug molecules under relatively mild conditions, often preserving chemical stability and promoting amorphous or crystalline dispersion depending on polymer-drug interactions [38, 40, 45]. As a result, electrospun fibers can support sustained, controlled, or burst release profiles tailored to therapeutic needs [35, 39, 40, 42, 45–48]. For chemotherapeutics, these properties are particularly advantageous, as they enable localized delivery, reduce systemic exposure, and may improve the stability of labile compounds [49, 50], making electrospinning attractive for intracranial drug-delivery strategies.

Direct-current (DC) electrospinning is the most commonly used configuration for fiber production [51, 52]. In DC systems a high voltage potential is applied between a spinneret and a grounded collector, forming a Taylor cone and driving a polymer jet toward the collector [51, 52]. As the jet elongates, it undergoes bending and whipping instabilities that can result in non-uniform deposition and reduced control over fiber morphology [51, 52]. In addition, DC electrospinning can be sensitive to solution properties, with challenges including charge accumulation, needle clogging, and limitations in incorporating high concentrations of active materials. These factors can complicate scale-up and hinder consistent drug incorporation, particularly for small-molecule chemotherapeutics [33]. Despite widespread use of DC electrospinning for drug-loaded nanofibers [46, 53], challenges remain in achieving efficient drug incorporation and predictable release behavior for chemically unstable therapeutics such as dacarbazine. Quantitative evaluation of drug loading and release is therefore essential for validating the performance of these systems [54–56].

**Fig. 1:**
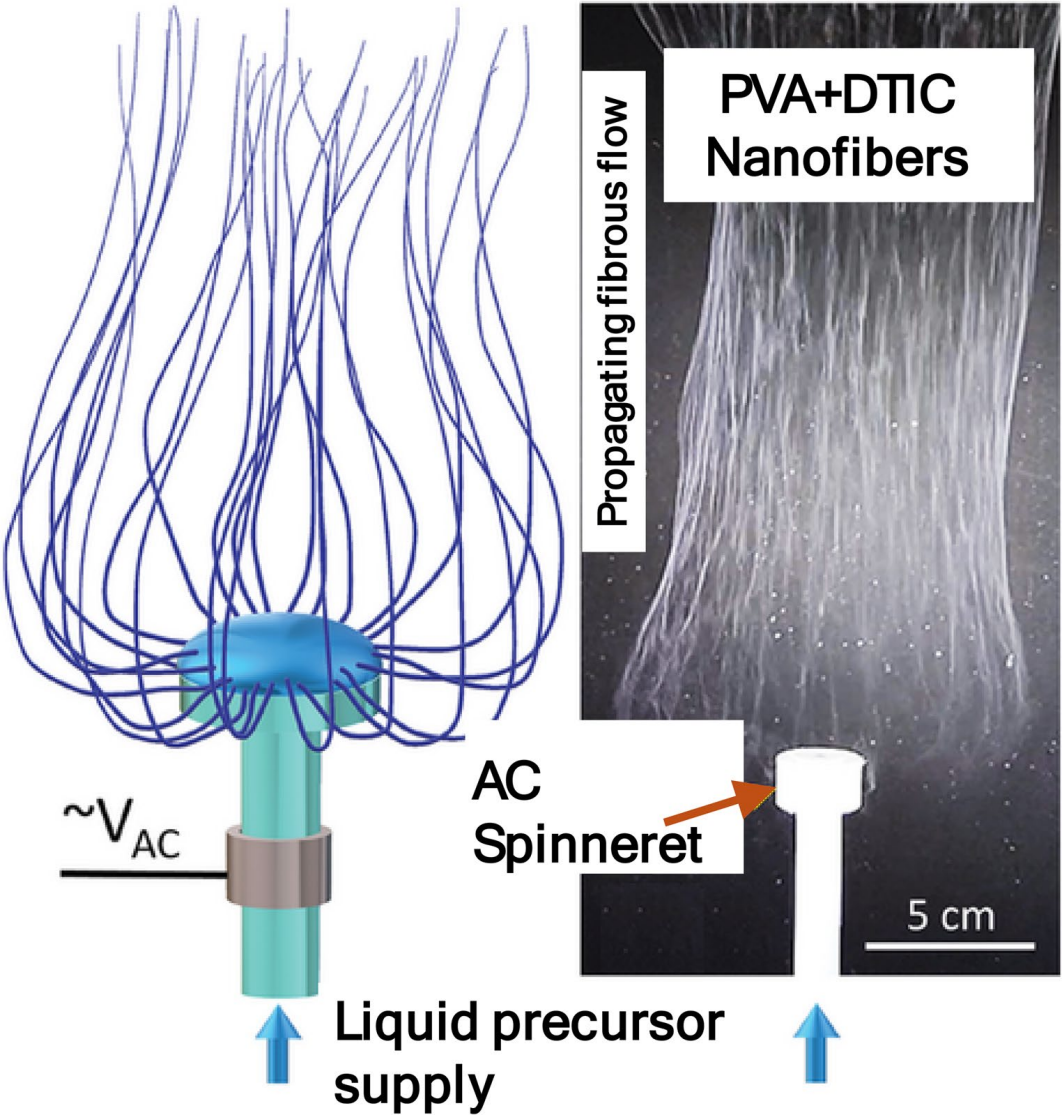
A schematic of an AC electrospinning setup used to develop the PVA and PVA-DTIC nanofibers in this work. (Adapted from Reference [59])

Electrospinning strategies based on alternating current (AC) fields have recently emerged as promising alternatives to conventional DC approaches [57–59]. Figure 1 shows a schematic of a novel AC electrospinning setup used for this work [60]. AC electrospinning introduces additional tunable parameters such as field frequency and waveform and reduces net charge accumulation through periodic charge recombination, enabling more stable jet behavior and improved fiber uniformity [57–59]. AC electrospun fibers can be produced at rates up to ~ 50 ml/hr, approximately two orders of magnitude greater than conventional DC systems, while maintaining fiber continuity [58, 59, 61]. The reduced sensitivity to charge accumulation also improves formulation flexibility, enabling more uniform incorporation of active materials at higher loading levels [51, 57–59, 61]. These characteristics make AC electrospinning particularly well-suited for aqueous or polar precursor systems, where DC processes often require extensive optimization. Accordingly, AC electrospinning was selected for fabricating DTIC-loaded PVA nanofibers to leverage its high productivity, reduced fiber charging, and compatibility with aqueous drug-polymer solutions.

Despite increasing interest in localized chemotherapeutic delivery, few studies have examined the solid-state stabilization or incorporation of DTIC into polymeric nanofibers. DTIC’s susceptibility to hydrolysis and photodegradation, short plasma half-life, and poor blood-brain barrier permeability highlight the need for formulations that protect the drug and enable localized intracranial delivery [62–64]. Although PVA nanofibers have been extensively studied for encapsulating small-molecule therapeutics [36], the behavior of DTIC within such matrices remains largely uncharacterized. Similarly, while AC electrospinning offers advantages for producing high-quality fibers from aqueous drug-polymer solutions [65–67], its application to chemotherapeutic systems remains limited [68, 69]. These gaps underscore the need for materials-focused investigations into the fabrication and solid-state properties of DTIC-loaded PVA nanofibers.

Based on prior reports demonstrating reduced fiber charging, improved jet stability, and higher production rates in AC electrospinning [57, 58, 60], we hypothesize that AC electrospinning can enable the fabrication of drug-loaded nanofibers with improved structural uniformity and controlled drug incorporation suitable for localized delivery applications.

The objective of this work is to develop and characterize DTIC-loaded PVA nanofibers fabricated using alternating-current electrospinning. Specifically, this study examines the influence of DTIC incorporation on fiber morphology, polymer-drug interactions, and solid-state structure using X-ray diffraction (XRD), Fourier-transform infrared spectroscopy (FT-IR), differential scanning calorimetry (DSC), scanning electron microscopy (SEM), and transmission electron microscopy (TEM). Encapsulation efficiency (EE) and drug release behavior are also evaluated using Ultraviolet-visible spectroscopy (UV-Vis).

## Experimental Sections

### Alternating Current Electrospinning of Nanofibers

Polyvinyl alcohol (PVA; 88,000 MW) solutions were prepared at a concentration of 8.3% w/v by dissolving as-received PVA powder in deionized water and ethanol under continuous stirring at 95 °C until fully solubilized. The solution was then allowed to cool to room temperature prior to drug incorporation. All steps involving DTIC were performed under low-light conditions, and containers were made of amber to minimize photodegradation.

DTIC was weighed separately and added to the cooled PVA solution to prepare formulations with PVA: DTIC mass ratios of 2.5:1, 5:1, 7.5:1, and 10:1. Each mixture was stirred until visually homogeneous which typically took roughly 12–18 h. No additional pH adjustment or organic solvents were used. A neat PVA solution (8.3% w/v) was prepared using the same procedure and served as the control formulation for fiber fabrication.

DTIC-loaded PVA nanofibers were produced using a custom-designed alternating-current (AC) electrospinning system developed by TruSpin Nanomaterial Innovation [60]. The apparatus employs a high-voltage AC electric field and a free-surface electrode configuration to generate continuous nanofiber plumes, consistent with previously reported AC electrospinning and alternating field electrospinning methods [59]. In this configuration, fiber formation is driven by periodic electric-field oscillations rather than a constant potential difference, enabling reduced net fiber charging and collectorless deposition.

Polymer–drug precursor solutions were introduced into the AC field at controlled flow conditions, and fibers were allowed to deposit onto a rotating, non-conductive drum collector positioned within the region of stable plume formation. Environmental conditions during spinning were maintained at controlled temperature and humidity ranges to ensure reproducible fiber morphology. All formulations were processed under low-light conditions to minimize DTIC photodegradation.

### Spectroscopic and Crystallographic Characterization

X-ray diffraction (XRD) was used to examine the structures of the constituents within the nanofiber. XRD characterization was performed using a Panalytical Empyrean multipurpose X-ray diffractometer operated at 45 kV and 40 mA with Cu Kα radiation (λ = 1.5406 Å). The diffraction patterns were used to identify crystalline features and to confirm the absence of any newly synthesized compounds in the nanofiber.

In addition, Fourier transform infrared (FT-IR) spectroscopy was used to analyze the various bonds in the constituents in the nanofiber. FT-IR spectra of the constituents in the nanofibers were collected in attenuated total reflection (ATR) mode using an infrared spectrophotometer (Bruker, USA). Each spectrum was recorded with 32 scans over the wavenumber range of 4000–400 cm⁻¹.

### Thermal Analysis

DSC was conducted with a DSC-250 calorimeter (TA Instruments, New Castle, DE) for all nanofibers. AC Electrospun Nanofibers and neat PVA and DTIC powder samples weighing 6–13 mg were tested in a 40–250 °C temperature range at a ramp rate of 10 °C/min for heating under the nitrogen gas purging rate of 50 ml/min. Calorimetric curves were analyzed using the TA Instruments Trios Software (TA Instruments, New Castle, DE) to obtain thermal properties of the nanofibers.

### Morphological Analysis

SEM images were acquired with a Quanta FEG 650 (Thermo Fisher Scientific) scanning electron microscope. Prior to imaging, the samples were prepared with Au-Pd sputter coating to promote surface conductivity and avoid charging. Because the electron beam with a high voltage can cause local melting of the sample due to the low melting temperature of the PVA matrix in the nanofiber, a low voltage of 5 kV was used for imaging. Uniformity of DTIC distribution in the nanofiber was evaluated using transmission electron microscopy (JEOL TEM-1400 Plus, Acceleration voltage 120 kV).

### Drug Encapsulation Efficiency

Encapsulation efficiency (EE) of dacarbazine within electrospun PVA nanofibers was determined using Ultraviolet-visible spectroscopy (UV-Vis) following complete dissolution of the fiber mats. Nanofiber samples from each formulation (PVA: DTIC ratios of 2.5:1, 5:1, 7.5:1, and 10:1) were collected and accurately weighed (~ 10–20 mg per sample). Each sample was dissolved in 10 mL of dimethyl sulfoxide (DMSO) under gentle vortexing and incubation for approximately 2 h to ensure complete polymer dissolution and drug extraction.

The resulting solutions were further diluted (1:100) in DMSO to bring absorbance values within the linear range of the calibration curve. Absorbance measurements were recorded using a UV-Vis spectrophotometer (Beckman DU-800 Spectrophotometer) at 327 nm, with baseline correction performed at 397 nm to account for background contributions. A standard calibration curve of dacarbazine in DMSO was generated over a relevant concentration range and used to determine drug concentration in each sample.

Encapsulation efficiency was calculated using the following equation:

$$EE(\%)=(\frac{Measured\;Drug\;Content}{Theoretical\;Drug\;Content})\times100$$

All measurements were performed in triplicate, and results are reported as mean ± standard deviation.

### Drug Release Analysis

In vitro release of dacarbazine from electrospun PVA nanofibers was evaluated in phosphate-buffered saline (PBS) at 37 °C (pH ~ 6.8). Nanofiber samples from the 2.5:1 PVA: DTIC formulation were selected for release studies based on their favorable morphology and encapsulation efficiency. Samples (approximately 20–30 mg) were immersed in 20 mL of PBS and maintained under static conditions at 37 °C.

At predetermined time points (5 min, 15 min, 30 min, 1 h, 2 h, 4 h, 8 h, 12 h, 24 h, 48 h, 72 h, and 120 h), 1 mL aliquots of the release medium were withdrawn and replaced with an equal volume of fresh PBS to maintain sink conditions. Collected samples were diluted (1:100) with PBS prior to analysis.

Dacarbazine concentration was quantified using UV-Vis spectroscopy (Beckman DU-800 Spectrophotometer) at 327 nm with baseline correction at 397 nm. Baseline correction using dual-wavelength analysis (327/397 nm) was employed to minimize interference from background absorbance. A calibration curve generated in PBS was used to determine drug concentration in the release medium. All measurements were performed in triplicate.

Due to variability in fiber mass and potential matrix effects influencing absolute concentration values, release data were normalized to the maximum observed release to enable comparison of relative release behavior across time points.

### Statistical Analysis

All data are presented as mean ± standard deviation (SD) unless otherwise noted. Data processing, plotting, and analysis were performed using OriginPro (OriginLab Corporation, Northampton, MA, USA).

## Results and Discussions

### Alternating Current Electrospinning Results

Dacarbazine-loaded PVA nanofibers were successfully fabricated using alternating-current (AC) electrospinning across all tested PVA: DTIC ratios (2.5:1, 5:1, 7.5:1, and 10:1). The AC electrospinning process enabled stable jet formation and continuous fiber production without the need for excessively high voltages or complex parameter optimization. Unlike conventional direct-current (DC) electrospinning, which is often limited by charge accumulation and jet instability, the AC process produced consistent fiber deposition with minimal bead formation under optimized conditions. This stability is attributed to the oscillating electric field, which reduces net charge buildup and allows for more uniform fiber formation. Qualitatively, the resulting fiber mats exhibited uniform morphology and consistent coverage across the collector surface. No visible phase separation or macroscopic drug aggregation was observed during fabrication, indicating that dacarbazine was well incorporated into the spinning solution prior to fiber formation. These observations demonstrate that AC electrospinning provides a robust and reproducible method for fabricating drug-loaded nanofibers, establishing the foundation for subsequent structural, encapsulation, and release analyses.

### Crystallographic and Spectroscopic Results

**Fig. 2:**
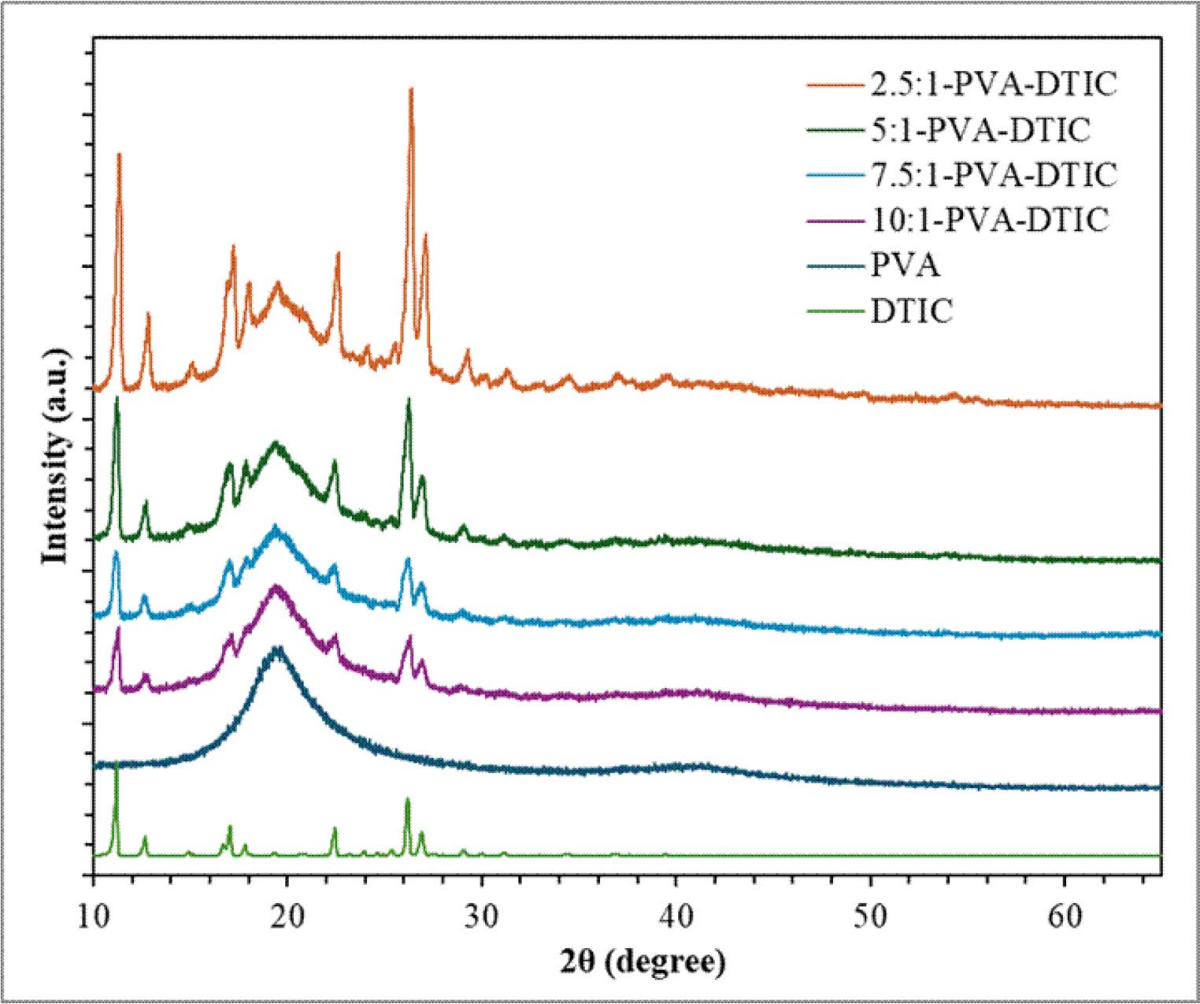
XRD spectra of pure PVA fiber, pure DTIC, and of PVA-DTIC nanofibers with different PVA: DTIC ratios, e.g., 2.5:1, 5:1, 7.5:1, and 10:1

XRD spectrum of pure PVA fiber and the PVA-DTIC fibers with different PVA: DTIC ratios are presented in Fig. 2. Pure PVA fiber shows a broad diffraction peak at approximately 2θ = 19–20°, indicating its semi-crystalline structure, while DTIC exhibits sharp peaks in the range of 10–30°, reflecting its high crystallinity. The nanofiber samples display characteristic peaks from both components. The intensity of DTIC peaks increases with increasing DTIC content, whereas the PVA amorphous halo becomes less pronounced. No new diffraction peaks are observed, suggesting that no new crystalline phase is formed in the nanofiber and DTIC remains individualistic in the PVA-DTIC mixture.

**Fig. 3:**
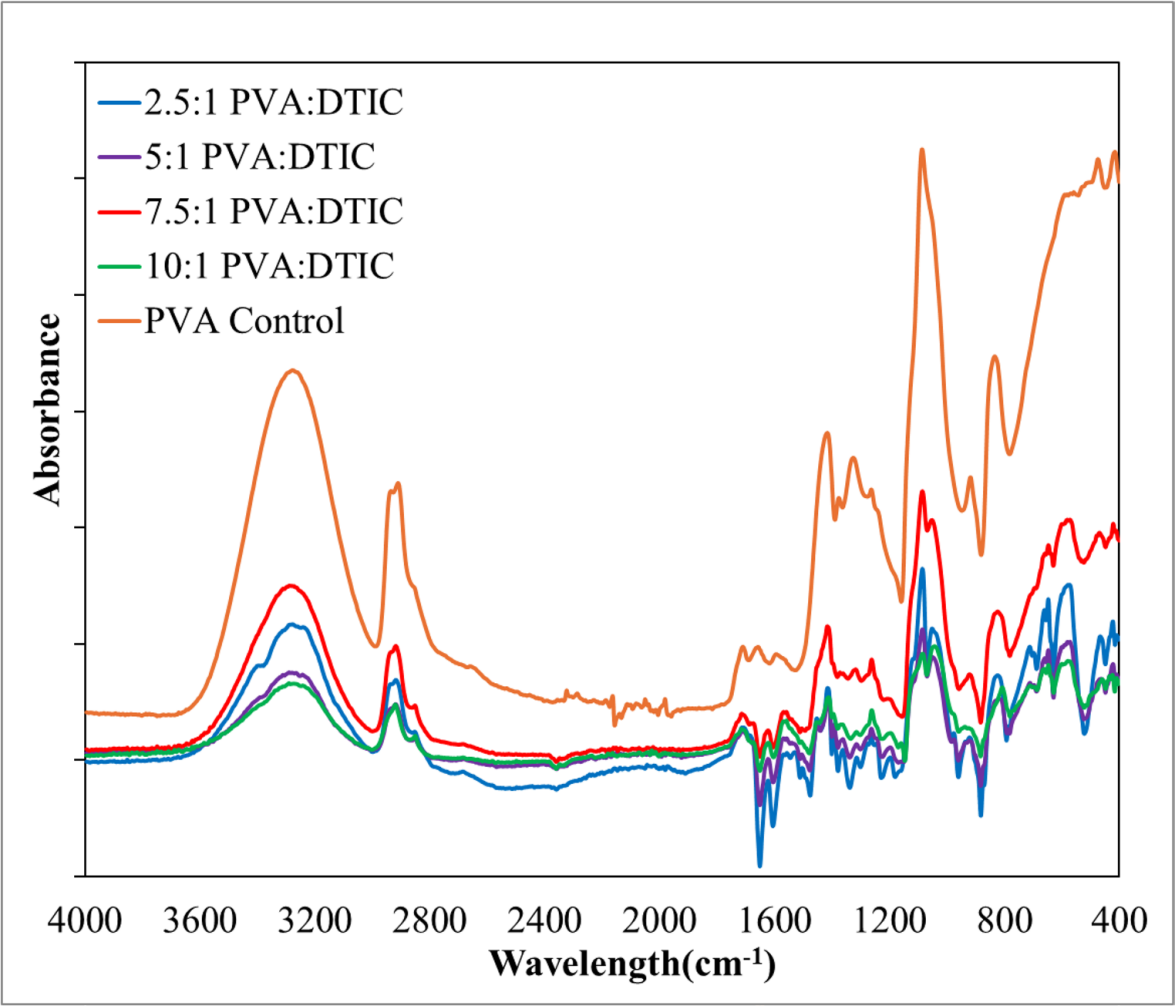
Superimposed FTIR spectrum of pure PVA nanofiber and PVA-DTIC nanofibers with different PVA: DTIC ratios, e.g., 2.5:1, 5:1, 7.5:1, and 10:1

The FTIR spectra of pure PVA fiber and nanofibers with different PVA: DTIC ratios are shown in Fig. 3. The broad absorption band around 3200–3500 cm⁻¹ corresponds to O–H stretching of PVA. Peaks near 2900 cm⁻¹ are attributed to C–H stretching vibrations. Characteristic absorption bands in the range of 1700–1000 cm⁻¹ arise from DTIC-related functional groups and PVA backbone vibrations. Changes in peak intensity with increasing DTIC content indicate the successful incorporation of DTIC into the PVA matrix, while no new absorption bands are observed, suggesting no new chemical bonds are formed. The characteristic absorption peaks of PVA and DTIC remain unchanged, and no additional peaks are observed. This confirms that no chemical reaction occurred between these two constituents. Therefore, AC electrospinning can be used to produce nanofibers without affecting the chemical stability of DTIC.

### Thermal Analysis Results

**Fig. 4:**
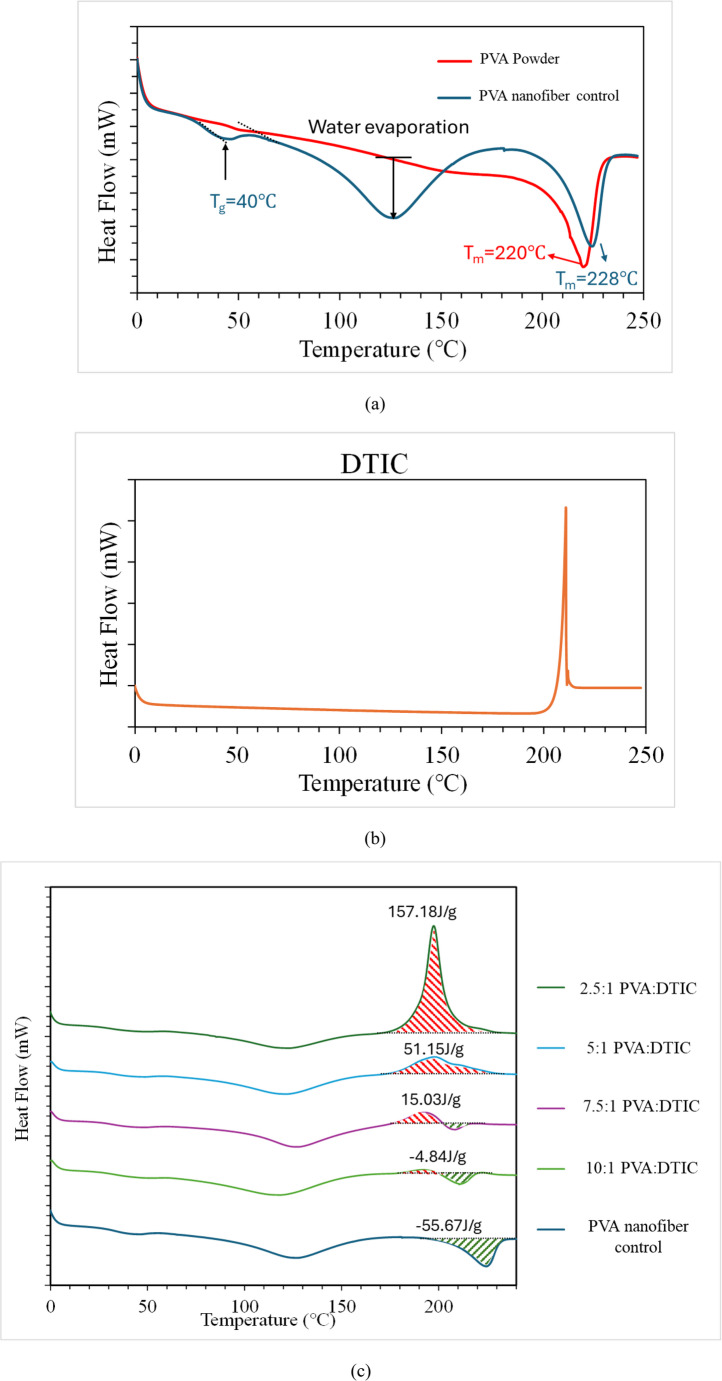
(**a**) DSC curves of raw PVA in powder form and AC electrospun PVA nanofibers. Due to the presence of residual solvents, the PVA nanofiber shows a pronounced endothermic peak associated with solvent evaporation; (**b**) DSC curve of pure DTIC exhibits an exothermic peak at around 200 °C; (c) DSC curves of AC electrospun nanofibers with different DTIC contents

Figure 4 shows the DSC curves for as receive pure PVA powder, electrospun pure PVA nanofiber, and the PVA-DTIC nanofibers. Pure PVA shows a broad thermal transition associated with its semi-crystalline structure, while DTIC exhibits a sharp endothermic peak near its melting temperature, indicating its high crystallinity. The nanofiber samples display combined thermal features of both components. Changes in peak shape and intensity with varying DTIC content indicate altered thermal behavior of PVA due to DTIC incorporation, while no additional thermal transitions are observed, suggesting no new phase or compound formation. The exothermic peak observed at around 200 °C in the nanofiber DSC curves increases with increasing DTIC content. This result further implies that the nanofibers can achieve a high DTIC loading, with a maximum DTIC: PVA ratio of 2.5:1. These results suggest that the nanofibers possess high DTIC content and exhibit good drug-loading capacity.

### Morphological Results

**Fig. 5:**
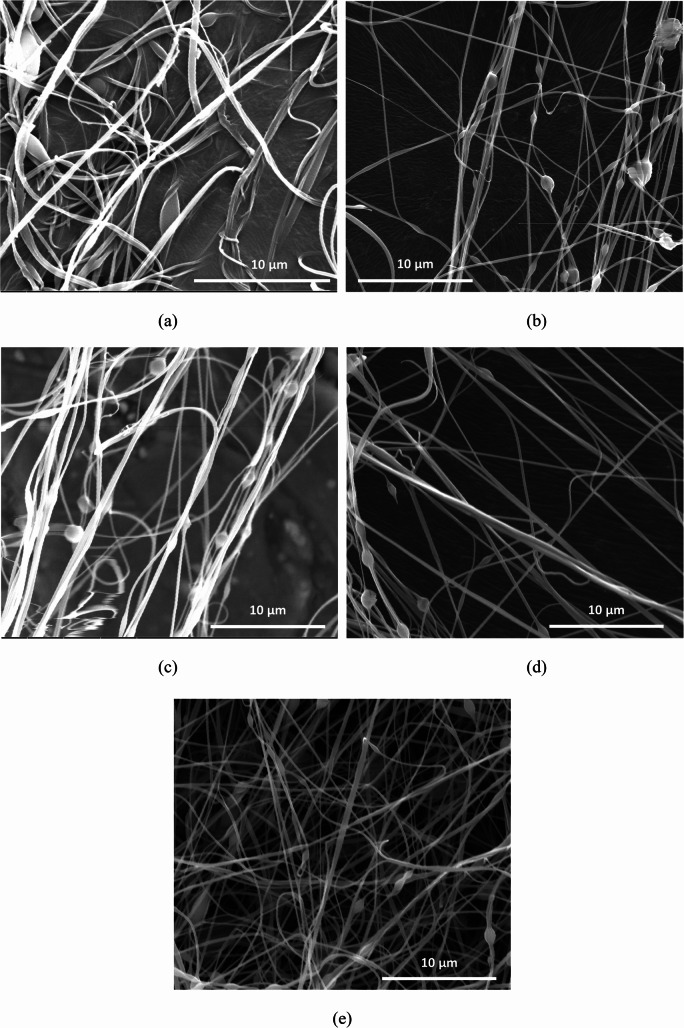
SEM images of nanofibers with different PVA:DTIC ratios: (**a**) pure PVA fiber, (**b**) 2.5: 1 ratio; (**c**) 5:1 ratio; (**d**) 7.5:1 ratio; and (**e**) 10:1 ratio

SEM images of pure PVA nanofibers and PVA-DTIC nanofibers with different PVA: DTIC ratios, e.g., 2.5:1, 5:1, 7.5:1, and 10:1, are shown in Fig. 5. Those fibers show similar morphologies. Although the sizes among individual nanofibers vary, it seems that individual nanofiber possess consistent size along the fiber length. In addition, minimal beads are noticed in all the nanofibers with different PVA: DTIC ratios. Bead formation is generally undesirable in electrospun drug-loaded nanofibers [45, 57], as beads can act as localized reservoirs with altered surface area and drug concentration, leading to non-uniform material properties and unpredictable drug distribution. In the present study, the use of ACES, combined with appropriate solution formulation, resulted in uniform fibrous morphologies with no significant bead formation observed across DTIC-loaded compositions as compared to PVA nanofibers produced using DC electrospinning [54]. This suggests that the ACES approach effectively mitigates jet instabilities that commonly contribute to bead formation, enabling consistent nanofiber fabrication suitable for localized drug delivery applications. 

**Fig. 6:**
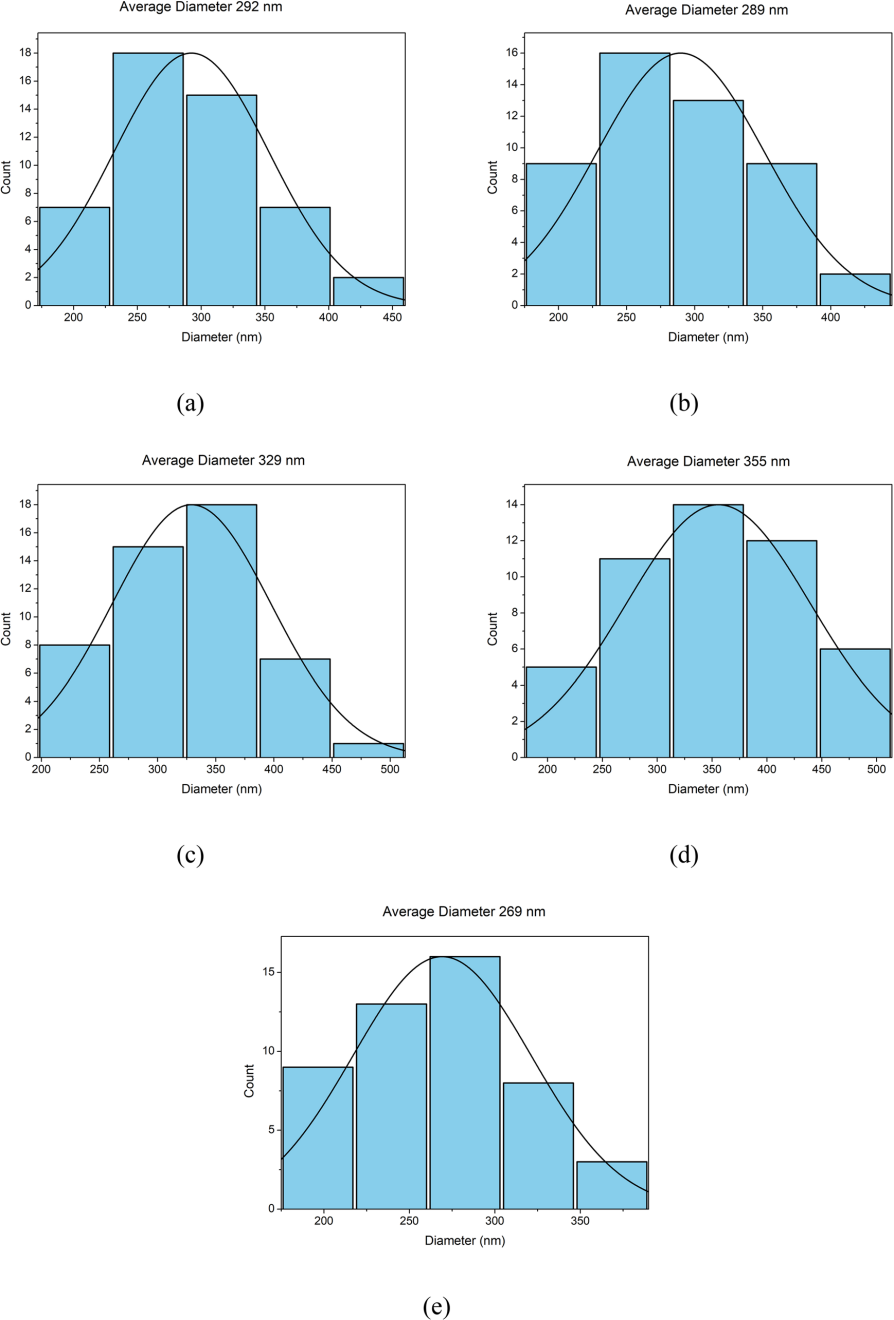
Diameter measurements of nanofibers with different PVA:DTIC ratios: (**a**) pure PVA fiber, (**b**) 2.5: 1 ratio; (**c**) 5:1 ratio; (**d**) 7.5:1 ratio; and (**e**) 10:1 ratio

Quantitative analysis of fiber diameters was performed using SEM images to evaluate the influence of DTIC loading on nanofiber morphology, Fig. 6. The PVA control fibers exhibited an average diameter of approximately 300 nm, while DTIC-loaded fibers displayed mean diameters ranging from approximately 276 to 376 nm depending on polymer-to-drug ratio. All drug-loaded formulations exhibited unimodal diameter distributions with no evidence of distinct secondary populations, indicating consistent fiber formation across compositions. The PVA control fibers exhibited a broader, partially bimodal diameter distribution, which is commonly reported for electrospun aqueous PVA systems. In contrast, DTIC-loaded formulations having unimodal diameter distributions across all ratios indicate more uniform fiber formation upon drug incorporation. Although a modest increase in average fiber diameter was observed at intermediate drug loadings, no significant bead formation or morphological instability was detected. These results demonstrate that ACES enables reproducible fabrication of uniform nanofibers over a range of dacarbazine concentrations.

**Fig. 7:**
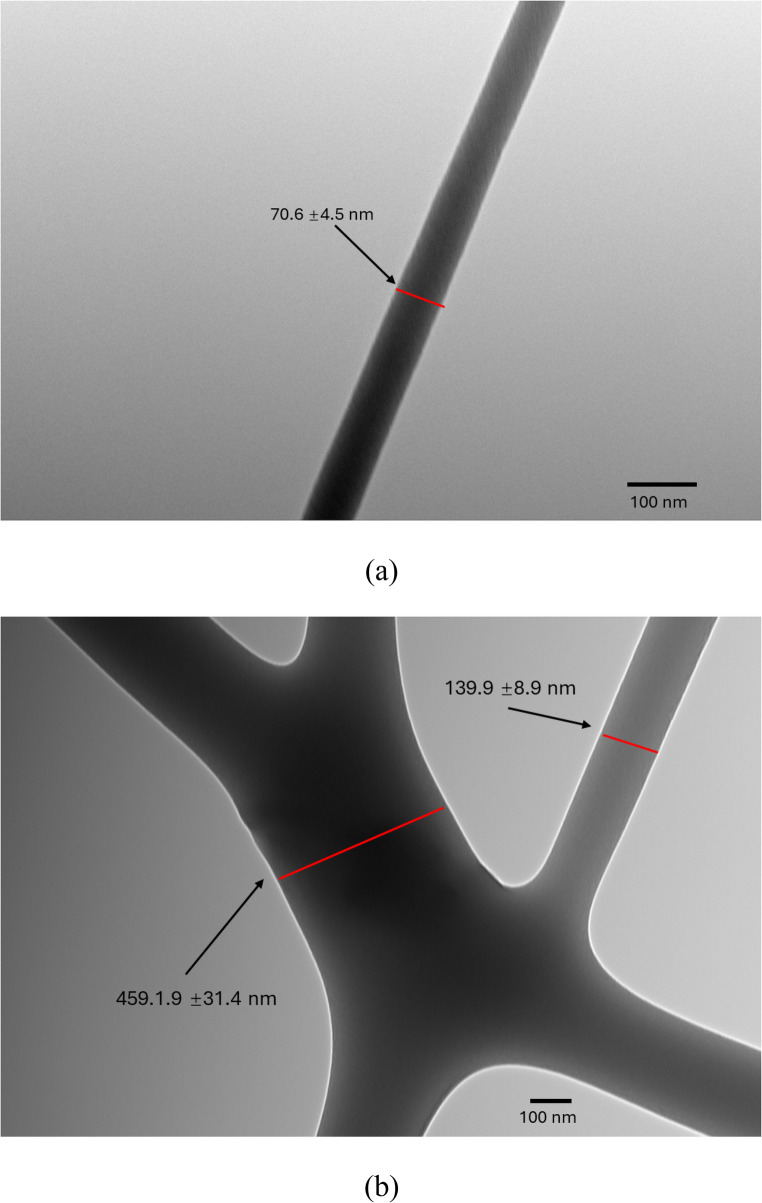
TEM images of AC electrospun (**a**) PVA nanofibers and (**b**) PVA-DTIC nanofibers with 5:1 PVA: DTIC ratio

TEM images of pure PVA nanofiber and 5:1 PVA-DTIC nanofibers are shown in Fig. 7. Fibers produced at different DTIC: PVA ratios exhibited diameters ranging from 70.6 ± 4.5 nm to 139.9 ± 8.9 nm. The largest fiber bundles showed an average diameter of 459 ± 31.4 nm. An individual fiber is shown in Fig. 7(a) which shows a consistent diameter. Although some fiber sections have features shown in Fig. 7(b), those imperfections can be improved by controlling fiber collection rate and humidity levels in future work.

Based on the SEM work performed on the nanofibers, it is confirmed that the PVA-DTIC nanofibers have smooth and defect free surfaces with minimal bead formation, which indicates that the incorporation of DTIC does not adversely affect jet stability or fiber solidification at concentrations of polymer-to-drug ratios as low as 2.5:1. The presence of crystalline DTIC peaks (Fig. 2) in XRD provides strong evidence for the individualistic DTIC in the nanofiber. Further, TEM imaging provided additional support for uniform drug distribution. TEM imaging revealed uniform internal contrast with no evidence of large-scale phase-separated domains or electron-dense inclusions within the nanofibers. This homogeneity is imperative for successful predictable release behavior of the drug as well as guaranteeing structural stability of the fibers.

### Encapsulation Efficiency Results

**Fig. 8:**
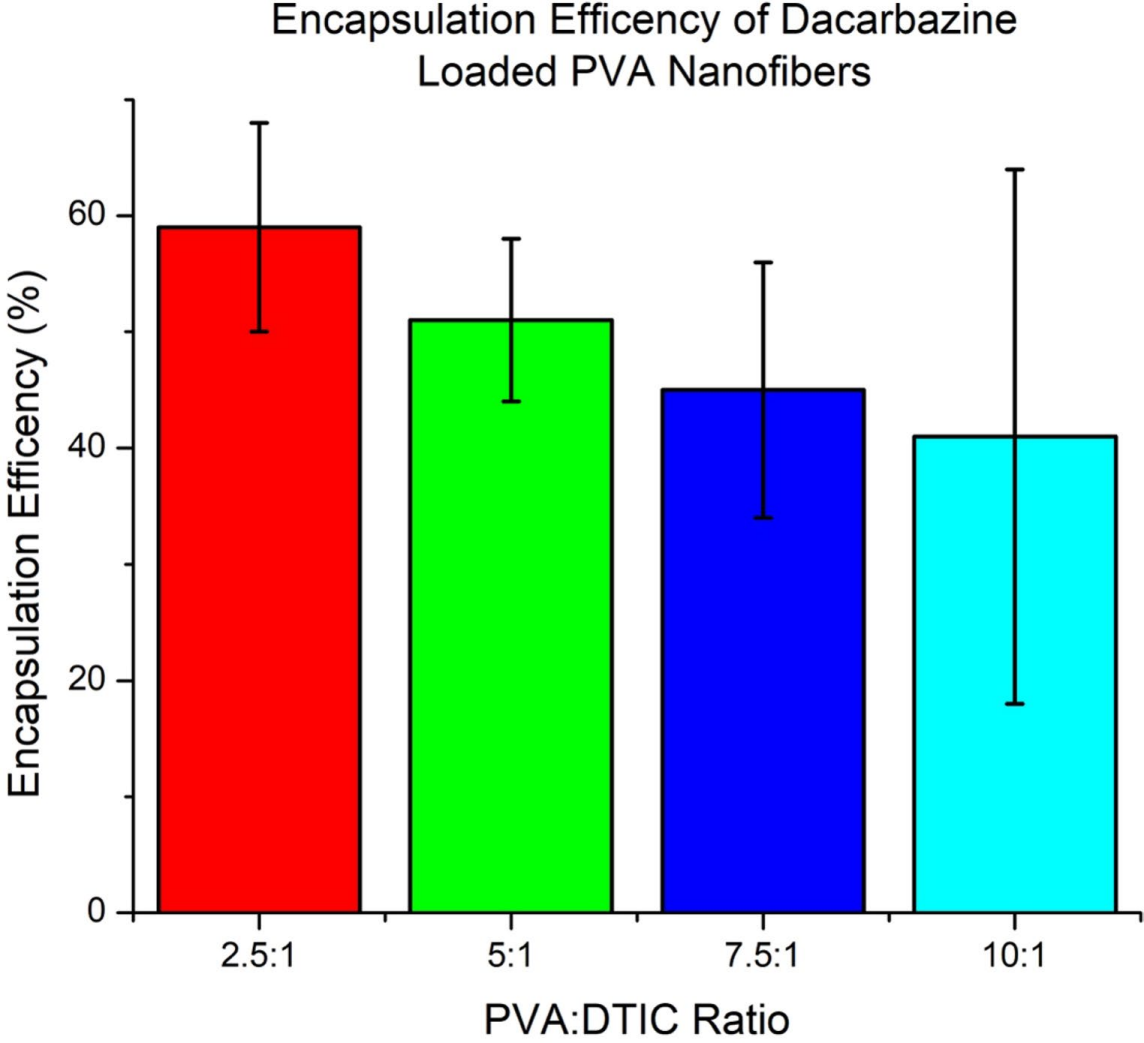
Encapsulation efficiency of dacarbazine-loaded PVA nanofibers at varying PVA: DTIC ratios. Drug content was quantified using UV-Vis spectroscopy following dissolution of nanofibers in DMSO. Data are presented as mean ± standard deviation (*n* = 3)

Encapsulation efficiency (EE) of dacarbazine within electrospun PVA nanofibers was quantified using UV-Vis spectroscopy following complete dissolution of the fiber mats (Fig. 8). The results demonstrate a clear dependence of drug incorporation on the PVA: DTIC ratio.

The highest encapsulation efficiency was observed for the 2.5:1 formulation, with values approaching ~ 60%, while progressively lower efficiencies were measured for increasing polymer-to-drug ratios, reaching approximately ~ 40% for the 10:1 formulation. This trend indicates that higher relative drug content promotes more efficient incorporation during the electrospinning process. This dataset can be used as a design tool for achieving desired EE as a function of PVA: DTIC ratio. Potentially higher EE can be achieved at ratios of 1.5:1 or 1:1 PVA: DTIC.

The observed decrease in EE with increasing polymer content is primarily due to reduced drug availability within the spinning solution. Overall, these results confirm that alternating-current electrospinning enables effective incorporation of dacarbazine into PVA nanofibers, with formulation-dependent control over drug loading.

### Drug Release Results

**Fig. 9:**
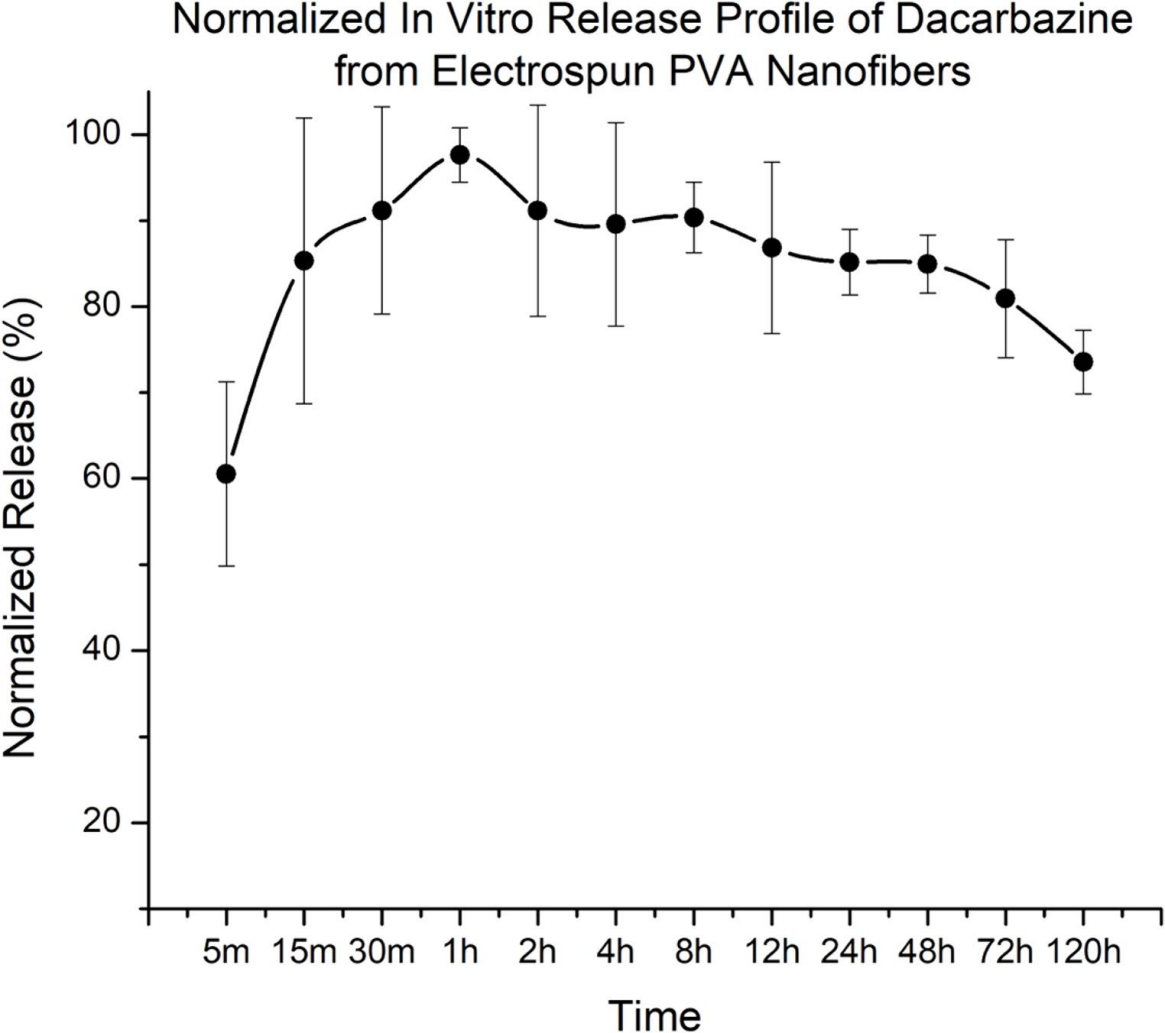
Normalized in vitro release profile of dacarbazine from electrospun PVA nanofibers (2.5:1 formulation) in phosphate-buffered saline at 37 °C. Drug concentration was quantified using UV-Vis spectroscopy and normalized to the maximum observed release to account for variability in fiber mass and measurement conditions. Data are presented as mean ± standard deviation (*n* = 3). An initial burst release is observed within the first hour, followed by sustained release behavior over 120 h

In vitro release studies were conducted to evaluate the release behavior of dacarbazine from electrospun PVA nanofibers in phosphate-buffered saline at 37 °C (Fig. 9). The release profile was normalized to the maximum observed release to account for variability in fiber mass and potential matrix effects influencing absolute concentration measurements.

An initial burst release was observed within the first hour, with approximately 60–100% of the normalized maximum release reached across replicates. This rapid release phase is attributed to surface-associated dacarbazine and drug located near the fiber-solution interface. Following the burst phase, the release profile exhibited a gradual decline in measured concentration over time. This trend is attributed to repeated sampling and replacement of the release medium, as well as potential dacarbazine instability under aqueous conditions, rather than a decrease in cumulative drug release. Despite this effect, the overall profile indicates sustained retention of dacarbazine within the nanofiber matrix over the duration of the study.

These results demonstrate that AC-electrospun PVA nanofibers are capable of incorporating dacarbazine and modulating its release behavior, supporting their potential as a platform for localized drug delivery. Further studies, including cytotoxicity, degradation, and in vivo evaluation, are warranted and will be the focus of future work.

## Conclusions

Dacarbazine-loaded PVA nanofibers with PVA-to-dacarbazine ratios of 2.5:1, 5:1, 7.5:1, to 10:1 were successfully developed using alternating-current electrospinning at an extremely high production rate. This work demonstrates that AC electrospinning can effectively incorporate dacarbazine into a nanofibrous matrix while maintaining fiber integrity and reproducibility. Compared to conventional direct-current (DC) electrospinning, which is often limited by charge accumulation and jet instability, AC electrospinning enables more stable fiber formation, thereby improving control over morphology and drug incorporation.

Structural characterization using XRD, FTIR, and DSC collectively indicates that dacarbazine is present in a non-crystalline, molecularly dispersed state within the PVA matrix. The absence of crystalline domains suggests that the drug is incorporated in an amorphous form, which is advantageous for improving apparent solubility and reducing the likelihood of recrystallization [70]. FTIR analysis further supports the presence of intermolecular interactions, including hydrogen bonding between PVA and dacarbazine, which may contribute to enhanced stabilization of the drug within the fibers. SEM and TEM analyses confirmed the formation of smooth, continuous fibers with homogenous internal structure, supporting uniform drug distribution throughout the matrix.

Encapsulation efficiency analysis revealed a clear dependence of drug loading on formulation composition, with the highest encapsulation efficiency observed for the 2.5:1 formulation and progressively lower efficiencies at higher polymer-to-drug ratios. This trend suggests that increased drug availability within the spinning solution promotes more effective incorporation, while higher polymer content may dilute the drug and increase the likelihood of loss during jet formation and fiber solidification. These findings confirm that AC electrospinning enables tunable drug loading based on formulation parameters.

In vitro release studies of the 2.5:1 formulation demonstrated an initial burst release within the first hour, followed by sustained release behavior over 120 h. The burst phase is attributed to surface-associated dacarbazine, while the subsequent release phase reflects diffusion of drug from within the nanofiber matrix [71–74]. Although normalized release profiles exhibited a gradual decrease in measured concentration over time, this effect is attributed to repeated sampling, dilution of the release medium, and potential dacarbazine instability in aqueous conditions, rather than a decrease in cumulative drug release. Together, these results indicate that the nanofiber system is capable of both rapid initial drug availability and prolonged retention, which are desirable characteristics for localized therapeutic delivery.

Taken together, the structural, encapsulation, and release data support the hypothesis that AC electrospinning provides a viable platform for the development of solid-state drug delivery systems. This approach is particularly relevant for intracranial drug delivery, where localized, sustained release systems are needed to overcome systemic delivery limitations. While the present study focuses on physicochemical characterization, several limitations should be acknowledged. Absolute drug loading values may be influenced by uncertainties in fiber mass measurement and UV-Vis quantification, and the aqueous stability of PVA remains a consideration as the incorporation of dacarbazine into the PVA matrix may also provide a protective effect by limiting its exposure to aqueous conditions, thereby reducing the rate of hydrolytic degradation relative to free drug in solution.

Future work will focus on improving system stability through crosslinking or polymer blending strategies, as well as evaluating biological performance through in vitro cytotoxicity, cellular uptake, and blood-brain barrier transport studies. These efforts will be critical for translating this platform toward clinical applications.

Overall, this work establishes the structural and physicochemical basis for using AC-electrospun PVA nanofibers as a localized dacarbazine delivery system. By enabling controlled drug incorporation and modulated release behavior, this platform shows strong potential for applications in intracranial drug delivery, where bypassing the blood-brain barrier remains a critical challenge.

## Data Availability

The data that support the findings of this study are available from the corresponding author upon reasonable request.
